# Asymmetric Synthesis of Tertiary α -Hydroxyketones by Enantioselective Decarboxylative Chlorination and Subsequent Nucleophilic Substitution

**DOI:** 10.3390/molecules25173902

**Published:** 2020-08-27

**Authors:** Mei Kee Kam, Akira Sugiyama, Ryouta Kawanishi, Kazutaka Shibatomi

**Affiliations:** Department of Applied Chemistry and Life Science, Toyohashi University of Technology, 1-1 Hibarigaoka, Tempaku-cho, Toyohashi 441-8580, Japan; meikeekam021@gmail.com (M.K.K.); sugiyama.akira.mb@tut.jp (A.S.); kawanishi.ryota.nc@tut.jp (R.K.)

**Keywords:** chlorination, S_N_2 reaction, asymmetric synthesis, organocatalyst, tertiary alcohols, α-hydroxyketones

## Abstract

Chiral tertiary α-hydroxyketones were synthesized with high enantiopurity by asymmetric decarboxylative chlorination and subsequent nucleophilic substitution. We recently reported the asymmetric decarboxylative chlorination of β-ketocarboxylic acids in the presence of a chiral primary amine catalyst to obtain α-chloroketones with high enantiopurity. Here, we found that nucleophilic substitution of the resulting α-chloroketones with tetrabutylammonium hydroxide yielded the corresponding α-hydroxyketones without loss of enantiopurity. The reaction proceeded smoothly even at a tertiary carbon. The proposed method would be useful for the preparation of chiral tertiary alcohols.

## 1. Introduction

**Scheme 1:**
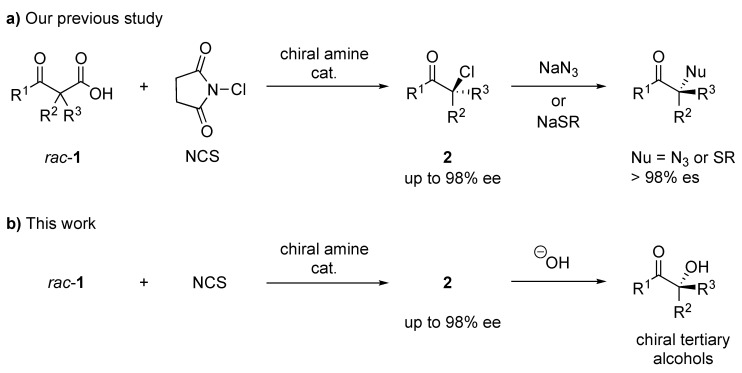
Asymmetric construction of a tetrasubstituted stereogenic center by enantioselective decarboxylative chlorination and subsequent nucleophilic substitution.

Chiral tertiary alcohols are important synthetic intermediates for the preparation of medicinally relevant compounds because numerous biologically active compounds contain the tertiary hydroxy moiety in their structure [1,2,3,4,5,6]. Using oxidative α-hydroxylation of carbonyl compounds is a useful method for introducing a hydroxy group onto a chiral carbon center. However, there are not many efficient methods for the catalytic asymmetric version of the α-hydroxylation of carbonyl compounds to form chiral tertiary alcohols, including the hydroxylation of enolates or enol ethers [7,8,9,10,11,12,13,14,15]. Recently, our research group developed a catalytic enantioselective decarboxylative chlorination of β-ketocarboxylic acids **1** using *N*-chlorosuccinimide (NCS) to afford tertiary α-chloroketones **2** with high enantioselectivity [16]. Moreover, the S_N_2 reaction of the resulting α-chloroketones proceeded smoothly with strong nucleophiles, such as sodium azide and alkyl thiols, even at a tertiary carbon center [16,17,18,19,20,21,22,23]. Inspired by these results, we successfully synthesized chiral tertiary α-hydroxyketones **3** by the enantioselective decarboxylative chlorination of β-ketocarboxylic acids and subsequent nucleophilic substitution with a hydroxide ion as the nucleophile (Scheme 1). The method would be a good alternative to the direct α-hydroxylation of simple ketones.

## 2. Results and Discussion

First, we attempted the nucleophilic substitution of tertiary α-chloroketones **2** with a hydroxide ion. The racemic form of tetralone-derived α-chloroketone **2a** was chosen as a model substrate. The reaction of **2a** with inorganic hydroxides led to the low conversion of the starting compound and yielded only trace amounts of the desired α-hydroxyketone **3a** (Table 1; entries 1 and 2). Although the use of tetrabutylammonium fluoride (TBAF) hydrate improved the conversion of the starting compound, **3a** was obtained in very poor yield, along with 38% of 2-allyl-1-naphthol (**4a**) as a by-product (entry 3). Compound **4a** was probably generated by the elimination of hydrogen chloride from **2a** and subsequent aromatization. Fortunately, **3a** was obtained in good yield (68%), along with **4a** in a 14% yield, when tetrabutylammonium hydroxide (TBAOH) was employed as the nucleophile (entry 4). Screening of various solvents revealed that acetonitrile was the best choice for the reaction.

**Figure 1:**
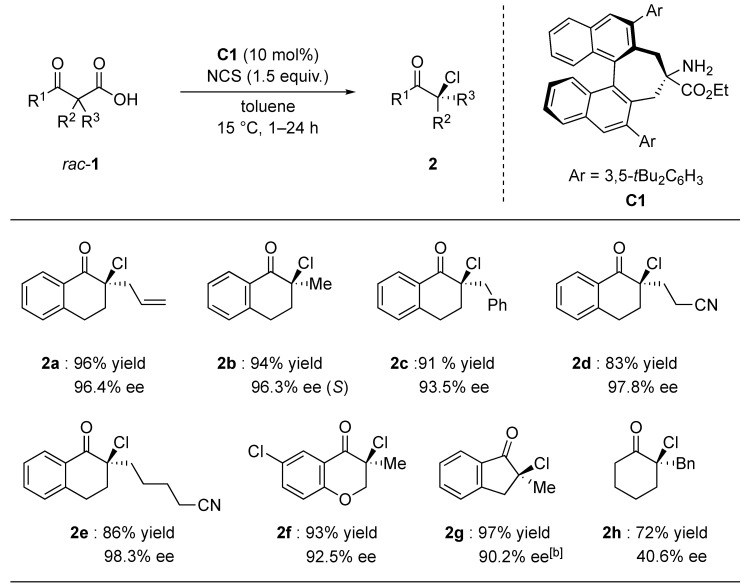
Synthesis of optically active α-chloroketones **2** ^[a]^ (^[a]^ Reactions were carried out with 10 mol% **C1** and 1.5 equiv. of NCS at ambient temperature in toluene (0.2 M), unless otherwise noted. ^[b]^ Reaction was carried out for 2 days at –20 °C).

**Figure 2:**
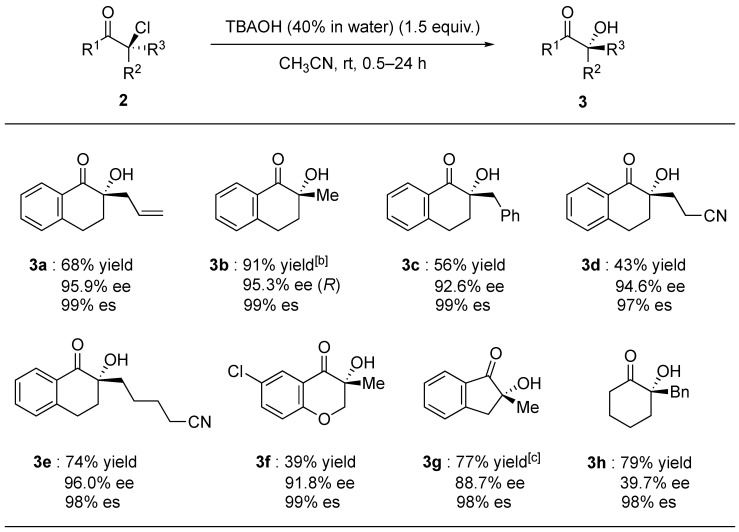
Substrate scope ^[a]^ (^[a]^ Reactions were carried out with 1.5 equiv. of TBAOH at ambient temperature in acetnitrile, unless otherwise noted. ^[b]^ Reaction was carried out at 0 °C. ^[c]^ Reaction was carried out with 3.0 equiv. of TBAOH at 0 °C. es: enantiospecificity).

Next, we prepared optically active tertiary α-chloroketones **2** for the asymmetric synthesis of α-hydroxyketone **3** by the enantioselective decarboxylative chlorination of β-ketocarboxylic acids **1** in the presence of chiral primary amine catalyst **C1** [16,24], according to our previous report [16]. As shown in Figure 1, a series of chiral α-chloroketones **2**, including tetralone derivatives **2a–2e**, 4-chromanone derivative **2f**, indanone derivative **2g**, and cyclohexanone derivative **2h**, were obtained in high yields, with moderate-to-high enantiopurity. It should be mentioned that the formation of Favorskii rearrangement product was not observed in the reaction of **2h** [22,25]. Then, we attempted the S_N_2 reaction of optically active **2** with tetrabutylammonium hydroxide under the optimized conditions. Accordingly, **2** was allowed to react with 1.5 equiv. of TBAOH (40% in water) in acetonitrile at ambient temperature. All the tested reactions proceeded smoothly to furnish the corresponding α-hydroxyketones **3** in high yields, without the loss of enantiopurity (Figure 2). The enantiospecificity of the reaction (ee of **3**/ee of **2**) was more than 97% in all cases.

**Scheme 2:**
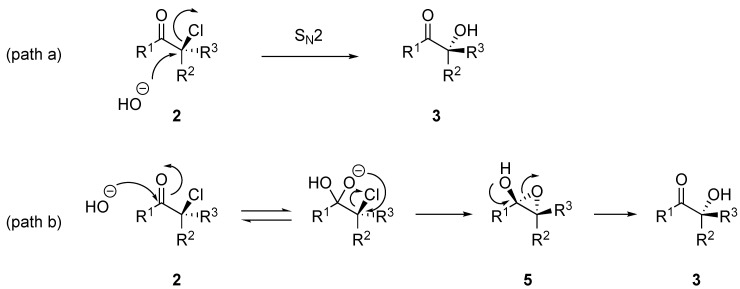
Two possible reaction pathways.

We propose two possible mechanisms for the nucleophilic substitution. The absolute configuration of **3b** obtained from (*S*)-**2b** was determined to be *R* by comparison of its specific rotation with the reported value [26]. Therefore, it is assumed that the reaction proceeded in the usual S_N_2 fashion (Scheme 2, path a). However, an alternative reaction mechanism involving the formation of an epoxide intermediate is proposed, where the hydroxide ion attacks the carbonyl carbon to form epoxide intermediate **5** via the release of a chlorine atom with inversion of stereochemistry. The subsequent regeneration of a carbonyl group affords **3** (path b) [27].

In summary, we achieved the asymmetric synthesis of α-hydroxyketones by the organocatalytic enantioselective decarboxylative chlorination of β-ketocarboxylic acids and subsequent nucleophilic substitution with TBAOH. Our method would be a good alternative to the direct α-hydroxylation of simple ketones. An advantage of our method is that the safe and easy-to-handle reagent NCS is employed as the formal oxidant.

## 3. Materials and Methods

### 3.1. General Methods

All non-aqueous reactions were carried out in dried glassware under an argon atmosphere and stirred using magnetic stir-plates. Thin-layer chromatography analyses were performed using pre-coated silica gel plates with a fluorescent indicator (F254) (Merck Millipore, Darmstadt, Germany). Visualization was accomplished by ultraviolet (UV) light (254 nm), phosphomolybdic acid, or *p*-anisaldehyde. Flash column chromatography was performed using silica gel 60 (mesh size 40–100) (Kanto Chemical Co., Inc., Tokyo, Japan). ^1^H and ^13^C nuclear magnetic resonance (NMR) spectra were recorded on a JNM-ECS400 (400 MHz ^1^H, 100 MHz ^13^C) or a JNM-ECX500 (500 MHz ^1^H, 126 MHz ^13^C) instrument (JEOL Ltd., Tokyo, Japan). Chemical shift values (δ) are reported in ppm (tetramethylsilane δ 0.00 ppm for ^1^H; residual chloroform δ 77.0 ppm for ^13^C). Direct analyses in real time (DART) mass (positive mode) analyses were performed on a JMS-T100TD time-of-flight mass spectrometer (JEOL Ltd.). Optical rotations were measured on a P-1030 digital polarimeter (JASCO Co., Ltd., Tokyo, Japan). Analytical high-performance liquid chromatography (HPLC) was performed on a PU1586 instrument with a MD-2018 plus diode array detector or PU1586 with a UV-1575 UV/Vis detector (JASCO Co., Ltd.) using a chiral column under the conditions described below. The enantiomeric purity of the compounds was determined by HPLC analyses using chiral stationary phase columns. ^1^H and ^13^C NMR spectra of compounds **1e**, **2e** and **3** and HPLC data of compounds **3** are available in Supplementary Materials.

### 3.2. Materials

Commercial grade reagents and solvents were used without further purification unless otherwise noted. Anhydrous toluene and tetrahydrofuran (THF) were purchased from Kanto Chemical Co., Inc. and used after purification by a Glass Contour solvent dispensing system (Pure Process Technology, Nashua, NH, USA). β-ketocarboxylic acids **1** and α-chloroketones **2** were prepared by the reported procedure [16].

### 3.3. Synthesis of β-ketocarboxylic Acid ***1e***

#### 3.3.1. *Tert*-Butyl 2-(2-cyanobutyl)-1-oxo-1,2,3,4-tetrahydronaphthalene-2-carboxylate

*tert*-Butyl-1-oxo-1,2,3,4-tetrahydronaphthalene-2-carboxylate (2.6 mmol) in THF (2.1 mL) was added to a stirred suspension of NaH (60% in oil, washed by hexane) (5.2 mmol, 2.0 equiv.) in THF (5.0 mL) at 0 °C, and the mixture was stirred at 0 °C for 1 h. Then, 5-Iodopentanenitrile (5.2 mmol, 2.0 equiv.) in THF (2.1 mL) was added to the mixture, and the mixture was stirred under reflux for 9 h. The reaction was quenched by adding sat. NH_4_Cl aq. at 0 °C, and then extracted with ethyl acetate. The organic layer was washed by brine, dried over Na_2_SO_4_, and concentrated under reduced pressure. The crude mixture was purified by flash column chromatography (hexane:ethyl acetate = 3:1) to yield the title compound as a yellowish oil (53% yield).

^1^H NMR (500 MHz, CDCl_3_): δ 8.01 (d, *J* = 7.6 Hz, 1H), 7.46 (dd, *J* = 7.6, 7.6 Hz, 1H), 7.31 (dd, *J* = 7.6 Hz, 7.6 Hz, 1H), 7.21 (d, *J* = 7.6 Hz, 1H), 3.12–3.06 (m, 1H), 2.92 (dt, *J* = 17.2, 4.6 Hz, 1H), 2.49 (dt, *J* = 13.4, 4.6 Hz, 1H), 2.38 (t, *J* = 7.3 Hz, 2H), 2.14–2.08 (m, 1H), 1.92–1.86 (m, 2H), 1.75–1.63 (m, 3H), 1.51–1.44 (m, 1H), 1.33 (s, 9H); ^13^C NMR (126 MHz, CDCl_3_): 195.8, 171.0, 142.6, 133.2, 132.5, 128.6, 127.6, 126.6, 119.6, 82.2, 57.6, 33.2, 31.2, 27.7, 26.0, 25.8, 23.9, 16.9; HRMS (DART): [M + NH_4_]^+^ calcd. for C_20_H_29_N_2_O_3_, 345.21782; found, 345.21778.

#### 3.3.2. 2-(4-Cyanobutyl)-1-oxo-1,2,3,4-tetrahydronaphthalene-2-carboxylic acid (**1e**)

Trifluoroacetic acid (16.8 mmol, 20 equiv.) was added to a stirred solution of *tert*-butyl 2-(2-cyanobutyl)-1-oxo-1,2,3,4-tetrahydronaphthalene-2-carboxylate (0.84 mmol) in dichloromethane (4.2 mL) at 0 °C, and the mixture was stirred at room temperature for 1 h. The reaction mixture was concentrated and then purified by flash column chromatography (hexane:diethyl ether = 4:1 to 1:2) to provide the title compound as a white solid (90% yield).

^1^H NMR (400 MHz, CDCl_3_): δ 8.06 (dd, *J* = 7.6 Hz, 1H), 7.52 (dd, *J* = 7.6, 7.6 Hz, 1H), 7.34 (t, *J* = 7.6Hz, 1H), 7.27–7.25 (m, 1H), 3.14–2.96 (m, 2H), 2.55–2.48 (m, 1H), 2.41–2.21 (m, 3H), 1.97–1.93 (m, 2H), 1.74–1.65 (m, 2H), 1.61–1.52 (m, 2H); ^13^C NMR (126 MHz, CDCl_3_): 196.9, 175.8, 143.3, 134.3, 131.0, 128.9, 128.3, 127.0, 119.4, 56.4, 33.1, 29.8, 25.5, 25.4, 23.9, 16.9; **HRMS** (DART): [M + NH_4_]^+^ calcd. for C_16_H_21_ N_2_O_3_, 289.15522; found, 289.15522.

### 3.4. Synthesis of α-chloroketone ***2e***

#### 5-(2-Chloro-1-oxo-1,2,3,4-tetrahydronaphthalen-2-yl)pentanenitrile (**2e**)

*N*-chlorosuccinimide (0.74 mmol, 1.5 equiv.) and 2-(4-cyanobutyl)-1-oxo-1,2,3,4-tetrahydro naphthalene-2-carboxylic acid (0.50 mmol) was added to a stirred solution of amine catalyst **C1** (0.05 mmol, 10 mol%) in toluene (25 mL) and the reaction mixture was stirred at 15 °C for 1 h. The crude product was purified by flash column chromatography (hexane:ethyl acetate = 3:1) to provide **2e** as a pale yellow solid (86% yield, 98% ee).

^1^H NMR (500 MHz, CDCl_3_): δ 8.09 (d, *J* = 7.6 Hz, 1H), 7.53–7.50 (m, 1H), 7.36–7.33 (m, 1H), 7.26 (d, *J* = 7.6 Hz, 1H), 3.37–3.31 (m, 1H), 2.94–2.90 (m, 1H), 2.48–2.21 (m, 5H), 2.15–2.10 (m, 1H), 1.78–1.68 (m, 3H), 1.59–1.54 (m, 1H); ^13^C NMR (126 MHz, CDCl_3_): 190.8, 142.7, 133.8, 129.7, 128.7, 128.6, 126.9, 119.3, 70.6, 37.3, 34.9, 25.6, 25.3, 23.3, 16.9; **HRMS** (DART): [M + H]^+^ calcd. for C_15_H_17_Cl_1_N_1_O_1_, 262.09987; found, 262.09992.

The enantiomeric purity of **2e** was determined by HPLC analysis (DAICEL CHIRALPAK IE-3 (0.46 cmϕ × 25 cm), hexane:2-propanol = 10:1, flow rate = 1.0 mL/min, retention time; 32.1 min (major) and 38.8 min (minor)).

### 3.5. Synthesis of α-hydroxyketones ***3***

#### 3.5.1. 2-Allyl-2-hydroxy-3,4-dihydronaphthalen-1(*2H*)-one (**3a**)

Tetrabutylammonium hydroxide (40% in water) (0.50 mmol, 1.5 equiv.) was added to a stirred solution of **2a** (0.33 mmol) in acetonitrile (1.7 mL), and the mixture was stirred at room temperature for 5 h. The reaction mixture was directly purified by flash column chromatography (hexane:ethyl acetate = 8:1) to provide **3a** as a pale yellow oil (68% yield, 95.9% ee) [13].

^1^H NMR (500 MHz, CDCl_3_): δ 8.02 (d, *J* = 7.6 Hz, 1H), 7.53 (dd, *J* = 7.6, 7.6 Hz, 1H), 7.35 (dd, *J* = 7.6, 7.6 Hz, 1H), 7.26 (d, *J* = 7.6 Hz, 1H), 5.92–5.84 (m, 1H), 5.17 (d, *J* = 10.3 Hz, 1H), 5.09 (dd, *J* = 17.0, 1.5 Hz, 1H), 3.83 (s, 1H), 3.14–3.07 (m, 1H), 2.99 (ddd, *J* = 18.0, 5.4, 2.3 Hz, 1H), 2.44 (dd, *J* = 14.1, 8.0 Hz, 1H), 2.38–2.33 (m, 2H), 2.17 (ddd, *J* = 13.0, 13.0, 5.4 Hz, 1H); ^13^C NMR (126 MHz, CDCl_3_): 201.0, 143.4, 134.1, 132.1, 130.1, 129.0, 128.0, 126.9, 119.1, 75.3, 40.3, 33.4, 26.1; **HRMS** (DART): [M + H]^+^ calcd. for C_13_H_15_O_2_, 203.10720; found, 203.10723.

[α]_D_^19^ + 3.94 (*c* = 1.35, CHCl_3_); The enantiomeric purity of **3a** was determined by HPLC analysis (DAICEL CHIRALPAK IC-3 (0.46 cmϕ × 25 cm), hexane:2-propanol = 9:1, flow rate = 1.0 mL/min, retention time; 9.4 min (minor) and 10.8 min (major)).

#### 3.5.2. (*R*)-2-Hydroxy-2-methyl-3,4-dihydronaphthalen-1(*2H*)-one (**3b**)

Tetrabutylammonium hydroxide (40% in water) (0.79 mmol, 1.5 equiv.) was added to a stirred solution of **2b** (0.53 mmol) in acetonitrile (2.7 mL) at 0 °C, and the reaction mixture was stirred at 0 °C for 24 h. The mixture was quenched by adding sat. NH_4_Cl aq. and extracted with ethyl acetate. The organic layer was washed by brine, dried over Na_2_SO_4_, and concentrated under reduced pressure. The crude product was purified by flash column chromatography (hexane:ethyl acetate = 9:1 to 4:1) to provide **3b** as a reddish oil (91% yield, 95.3% ee) [13].

^1^H NMR (400 MHz, CDCl_3_): δ 8.04 (d, *J* = 7.6 Hz, 1H), 7.53 (dd, *J* = 7.6, 7.6 Hz, 1H), 7.35 (dd, *J* = 7.6, 7.6 Hz, 1H), 7.26 (d, *J* = 7.6 Hz, 1H), 3.85 (s, 1H), 3.16–3.00 (m, 2H), 2.30–2.18 (m, 2H), 1.40 (s, 3H); ^13^C NMR (100 MHz, CDCl_3_): δ 201.8, 143.4, 134.1, 129.9, 129.0, 128.0, 126.9, 73.6, 35.8, 26.8, 23.9; **HRMS** (DART): [M + H]^+^ calcd. for C_11_H_13_O_2_, 177.09155; found 177.09165.

[α]_D_^26^ + 12.5 (*c* = 0.75, CHCl_3_); The absolute of **3b** was assigned by comparing its specific rotation with that of the same compound reported in the literature [26]. Lit. [α]_D_^20^ + 17.3 (*c* = 2.00, CHCl_3_) for 95% ee (*R*-configuration). The enantiomeric purity of **3b** was determined by HPLC analysis (DAICEL CHIRALPAK IA-3 (0.46 cmϕ × 25 cm), hexane:2-propanol = 24:1, flow rate = 0.7 mL/min, retention time; 17.7 min (major) and 19.6 min (minor)).

#### 3.5.3. 2-Benzyl-2-hydroxy-3,4-dihydronaphthalen-1(*2H*)-one (**3c**)

Tetrabutylammonium hydroxide (40% in water) (0.34 mmol, 1.5 equiv.) was added to a stirred solution of **2c** (0.22 mmol) in acetonitrile (1.1 mL), and the reaction mixture was stirred at room temperature for 7 h. The mixture was quenched by adding sat. NH_4_Cl aq. and extracted with ethyl acetate. The organic layer was washed by brine, dried over Na_2_SO_4_, and concentrated under reduced pressure. The crude product was purified by flash column chromatography (hexane:ethyl acetate = 8:1) to provide **3c** as a yellow solid (56% yield, 92.6% ee) [13].

^1^H NMR (400 MHz, CDCl_3_): δ 8.03–8.01 (m, 1H), 7.57 (dd, *J* = 7.6, 7.6 Hz, 1H), 7.39 (dd, *J* = 7.6, 7.6 Hz, 1H), 7.32–7.24 (m, 4H), 7.16–7.14 (m, 2H), 3.78 (s, 1H), 3.27 (ddd, *J* = 18.0, 12.5, 5.5 Hz, 1H), 3.07–3.01 (m, 1H), 3.00 (d, *J* = 13.7 Hz, 1H), 2.92 (d, *J* = 13.7 Hz, 1H), 2.28 (ddd, *J* = 13.4, 5.5, 2.1 Hz, 1H), 2.20 (m, 1H); ^13^C NMR (100 MHz, CDCl_3_): 201.0, 143.2, 135.3, 134.1, 130.3, 129.1, 128.1, 128.0, 127.1, 126.9, 76.0, 41.9, 33.8, 26.3; **HRMS** (DART): [M + H]^+^ calcd. for C_17_H_17_O_2_, 253.12285; found, 253.12289.

[α]_D_^26^ – 23.5 (*c* = 1.47, CHCl_3_); The enantiomeric purity of **3c** was determined by HPLC analysis (DAICEL CHIRALPAK IC-3 (0.46 cmϕ × 25 cm), hexane:2-propanol = 49:1, flow rate = 1.0 mL/min, retention time; 26.0 min (minor) and 29.2 min (major)).

#### 3.5.4. 3-(2-Hydroxy-1-oxo-1,2,3,4-tetrahydronaphthalen-2-yl)propanenitrile (**3d**)

Tetrabutylammonium hydroxide (40% in water) (0.24 mmol, 1.5 equiv.) was added to a stirred solution of **2d** (0.16 mmol) in acetonitrile (0.79 mL), and the reaction mixture was stirred at room temperature for 7 h. The reaction mixture was directly purified by open column chromatography (hexane:ethyl acetate = 3:1 to 1:1) to provide **3d** as a colorless solid (43% yield, 94.6% ee).

^1^H NMR (500 MHz, CDCl_3_): δ 8.02 (d, *J* = 7.6 Hz, 1H), 7.58 (dd, *J* = 7.6, 7.6 Hz, 1H), 7.38 (dd, *J* = 7.6, 7.6 Hz, 1H), 7.29 (d, *J* = 7.6 Hz, 1H), 3.94 (s, 1H), 3.17–3.04 (m, 2H), 2.58 (ddd, *J* = 16.8, 9.6, 6.5 Hz, 1H), 2.39–2.31 (m, 2H), 2.22 (ddd, *J* = 13.8, 12.6, 6.1 Hz, 1H), 2.12 (ddd, *J* = 14.1, 9.6, 6.1 Hz, 1H), 1.99 (ddd, *J* = 14.1, 9.6, 5.7 Hz, 1H); ^13^C NMR (126 MHz, CDCl_3_): 200.4, 143.0, 134.7, 129.5, 129.2, 128.2, 127.3, 119.4, 74.3, 33.8, 31.2, 26.2, 11.4; **HRMS** (DART): [M + H]^+^ calcd. for C_13_H_14_ N_1_O_2_, 216.10245; found, 216.10247.

[α]_D_^19^ + 35.5 (*c* = 0.57, CHCl_3_); The enantiomeric purity of **3d** was determined by HPLC analysis (DAICEL CHIRALPAK IB-3 (0.46 cmϕ × 25 cm), hexane:2-propanol = 10:1, flow rate = 1.0 mL/min, retention time; 18.3 min (major) and 21.0 min (minor)).

#### 3.5.5. 5-(2-Hydroxy-1-oxo-1,2,3,4-tetrahydronaphthalen-2-yl)pentanenitrile (**3e**)

Tetrabutylammonium hydroxide (40% in water) (0.30 mmol, 1.5 equiv.) was added to a stirred solution of **2e** (0.20 mmol) in acetonitrile (1.0 mL), and the reaction mixture was stirred at room temperature for 9 h. The reaction mixture was directly purified by open column chromatography (hexane:ethyl acetate = 3:1) to provide **3e** as a yellow oil (74% yield, 96.0% ee).

^1^H NMR (500 MHz, CDCl_3_): δ 8.01 (d, *J* = 7.6 Hz, 1H), 7.54 (dd, *J* = 7.6, 7.6 Hz, 1H), 7.36 (dd, *J* = 7.6, 7.6, 1H), 7.28–7.26 (m, 1H), 3.88 (s, 1H), 3.12–2.99 (m, 2H), 2.37–2.31(m, 3H), 2.17 (ddd, *J* = 13.4, 12.6, 6.1 Hz, 1H), 1.77–1.72 (m, 1H), 1.68–1.57 (m, 4H), 1.51–1.43 (m, 1H); ^13^C NMR (126 MHz, CDCl_3_): 201.6, 143.3, 134.2, 130.0, 129.1, 128.0, 127.0, 119.4, 75.3, 34.6, 33.8, 26.5, 25.5, 22.3, 17.1; **HRMS** (DART): [M + NH_4_]^+^ calcd. for C_15_H_21_N_2_O_2_, 261.16030; found, 261.16036.

[α]_D_^19^ + 37.9 (*c* = 1.18, CHCl_3_); The enantiomeric purity of **3e** was determined by HPLC analysis (DAICEL CHIRALPAK IA-3 (0.46 cmϕ × 25 cm), hexane:2-propanol = 10:1, flow rate = 1.0 mL/min, retention time; 22.7 min (major) and 25.4 min (minor)).

#### 3.5.6. 6-Chloro-3-hydroxy-3-methylchroman-4-one (**3f**)

Tetrabutylammonium hydroxide (40% in water) (0.21 mmol, 1.5 equiv.) was added to a stirred solution of **2f** (0.14 mmol) in acetonitrile (0.68 mL), and the reaction mixture was stirred at room temperature for 1 h. The reaction mixture was directly purified by open column chromatography (hexane:ethyl acetate = 5:1 to 1:1) to provide **3f** as a colorless oil (39% yield, 91.8% ee).

^1^H NMR (500 MHz, CDCl_3_): δ 7.84 (d, *J* = 2.7 Hz, 1H), 7.47 (dd, *J* = 8.8, 2.7 Hz, 1H), 6.96 (d, *J* = 8.8 Hz, 1H), 4.31, (d, *J* = 11.3 Hz, 1H), 4.20 (d, *J* = 11.3 Hz, 1H), 3.53 (s, 1H), 1.46 (s, 3H); ^13^C NMR (126 MHz, CDCl_3_): 195.5, 159.8, 136.5, 127.5, 126.8, 119.7, 119.0, 74.7, 70.6, 22.4; **HRMS** (DART): [M + H]^+^ calcd. for C_10_H_10_Cl_1_O_3_, 213.03185; found, 213.03190.

[α]_D_^20^ + 46.1 (*c* = 0.20, CHCl_3_); The enantiomeric purity of **3f** was determined by HPLC analysis (DAICEL CHIRALPAK AD-H (0.46 cmϕ × 25 cm), hexane:2-propanol = 49:1, flow rate = 1.0 mL/min, retention time; 23.4 min (major) and 30.6 min (minor)).

#### 3.5.7. 2-Hydroxy-2-methyl-2,3-dihydro-1*H*-inden-1-one (**3g**)

Tetrabutylammonium hydroxide (40% in water) (0.79 mmol, 3.0 equiv.) was added to a stirred solution of **2g** (0.26 mmol) in acetonitrile (1.3 mL) at 0 °C, and the reaction mixture was stirred at 0 °C for 18 h. The reaction mixture was directly purified by open column chromatography (hexane:ethyl acetate = 2:1) to provide **3g** as a colorless oil (77% yield, 88.7% ee) [13].

^1^H NMR (400 MHz, CDCl_3_): δ 7.79 (d, *J* = 7.6 Hz, 1H), 7.64 (dd, *J* = 7.6, 7.6 Hz, 1H), 7.45 (d, *J* = 7.6 Hz, 1H), 7.41 (dd, *J* = 7.6, 7.6 Hz, 1H), 3.28 (d, *J* = 16.8 Hz, 1H), 3.23 (d, *J* = 16.8 Hz, 1H), 2.81 (br, 1H), 1.45 (s, 3H); ^13^C NMR (100 MHz, CDCl_3_): δ 208.0, 151.2, 135.9, 133.5, 127.9, 126.8, 124.9, 77.4, 42.1, 25.7; **HRMS** (DART): [M + H]^+^ calcd. for C_10_H_11_O_2_, 163.07590; found, 163.07592.

[α]_D_^20^ + 38.8 (*c* = 1.1, CHCl_3_); The enantiomeric purity of **3g** was determined by HPLC analysis (DAICEL CHIRALPAK IC-3 (0.46 cmϕ × 25 cm), hexane:2-propanol = 9:1, flow rate = 0.7 mL/min, retention time; 22.6 min (minor) and 24.2 min (major)).

#### 3.5.8. 2-Benyl-2-hydroxycyclohexan-1-one (**3h**)

Tetrabutylammonium hydroxide (40% in water) (0.12 mmol, 1.5 equiv.) was added to a stirred solution of **2h** (0.082 mmol) in acetonitrile (0.41 mL), and the reaction mixture was stirred at room temperature for 1 h. The reaction mixture was directly purified by open column chromatography (hexane:ethyl acetate = 5:1) to provide **3h** as a colorless solid (79% yield, 39.7% ee) [28].

^1^H NMR (500 MHz, CDCl_3_): δ 7.29–7.19 (m, 5H), 3.86 (s, 1H), 3.14 (d, *J* = 13.8 Hz, 1H), 2.98 (d, *J* = 13.8 Hz, 1H), 2.70 (ddd, *J* = 14.1, 13.8, 6.1 Hz, 1H), 2.56–2.52 (m, 1H), 2.24–2.15 (m, 2H), 1.95–1.84 (m, 2H), 1.74–1.63 (m, 2H); ^13^C NMR (126 MHz, CDCl_3_): 213.2, 135.3, 130.0, 128.2, 126.9, 79.2, 43.2, 40.3, 38.5, 27.9, 22.8; **HRMS** (DART): [M + NH_4_]^+^ calcd. for C_13_H_20_N_1_O_2_, 222.14940; found, 222.14940.

[α]_D_^20^ – 33.3 (*c* = 0.58, CHCl_3_); The enantiomeric purity of **3h** was determined by HPLC analysis (DAICEL CHIRALPAK OJ-H (0.46 cmϕ × 25 cm), hexane:2-propanol = 10:1, flow rate = 1.0 mL/min, retention time; 17.4 min (minor) and 19.6 min (major)).

## Figures and Tables

**Table 1:**
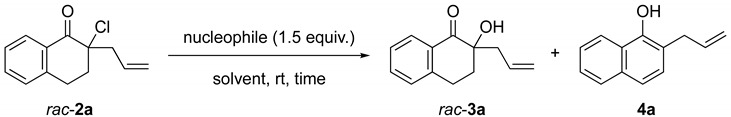
Optimization of reaction conditions ^[a]^.

Entry	Nucleophile	Solvent	Time (h)	Conv. (%) ^[b]^ of *rac*-2a	Yield (%) ^[c]^ of *rac*-3a	Yield (%) ^[c]^ of 4a
1	NaOH aq. (10 M)	CH_3_CN	5	19	1	1
2	KOH aq. (10 M)	CH_3_CN	5	10	4	3
3	TBAF·*n*H_2_O (1.0 M in THF)	CH_3_CN	5	61	1	38
4	TBAOH (40% in water)	CH_3_CN	5	99	68	14
5	TBAOH (40% in water)	THF	4	96	59	33
6	TBAOH (40% in water)	DMPU	45 min	100	48	39
7	TBAOH (40% in water)	DMF	2	100	52	27
8	TBAOH (40% in water)	toluene	4	33	22	5

^[a]^ Reactions were carried out with 1.5 equiv. of nucleophile at ambient temperature. ^[b]^ Calculated based on the number of moles of recovered **2a**. ^[c]^ Isolated yield. DMPU: *N*,*N′*-dimethylpropyleneurea.

## Supplementary Materials

The following are available online: ^1^H and ^13^C NMR spectra of compounds **1e**, **2e** and **3** and HPLC data of compounds **3**.

## References

[1] Liu Y.-L., Lin X.-T. (2019). Recent advances in catalytic asymmetric synthesis of tertiary alcohols via nucleophilic addition to ketones. Adv. Synth. Catal..

[2] Olack G., Morrison H. (1991). Formation and characterization of lumitetracycline-type photoproducts from members of the tetracycline family. J. Org. Chem..

[3] Lucas-Lopez C., Patterson S., Blum T., Straight A.F., Toth J., Slawin A.M.Z., Mitchison T.J., Sellers J.R., Westwood N.J. (2005). Absolute stereochemical assignment and fluorescence tuning of the small molecule tool, (–)-blebbistatin. Eur. J. Org. Chem..

[4] Matsuda H., Yoshida K., Miyagawa K., Asao Y., Takayama S., Nakashima S., Xu F., Yoshikawa M. (2007). Rotenoids and flavonoids with anti-invasion of HT1080, anti-proliferation of U937, and differentiation-inducing activity in HL-60 from *Erycibe expansa*. Bioorg. Med. Chem..

[5] Edwards M.G., Kenworthy M.N., Kitson R.R.A., Scott M.S., Taylor R.J.K. (2008). The telescoped intramolecular Michael/olefination (TIMO) approach to α-alkylidene-γ-butyrolactones: Synthesis of (+)-paeonilactone B. Angew. Chem. Int. Ed..

[6] Kusumi S., Nakayama H., Kobayashi T., Kuriki H., Matsumoto Y., Takahashi D., Toshima K. (2016). Total synthesis of aquayamycin. Chem. Eur. J..

[7] Masui M., Ando A., Shioiri T. (1988). New methods and reagents in organic synthesis. 75. asymmetric synthesis of α-hydroxy ketones using chiral phase transfer catalysts. Tetrahedron Lett..

[8] Morikawa K., Park J., Andersson P.G., Hashiyama T., Sharpless K.B. (1993). Catalytic asymmetric dihydroxylation of tetrasubstituted olefins. J. Am. Chem. Soc..

[9] Momiyama N., Yamamoto H. (2003). Catalytic enantioselective synthesis of α-aminooxy and α-hydroxy ketone using nitrosobenzene. J. Am. Chem. Soc..

[10] Kawasaki M., Li P., Yamamoto H. (2008). Enantioselective O-nitroso aldol reaction of silyl enol ethers. Angew. Chem. Int. Ed..

[11] Yanagisawa A., Takeshita S., Izumi Y., Yoshida K. (2010). Enantioselective nitroso aldol reaction catalyzed by quinoxP*·silver(I) complex and tin methoxide. J. Am. Chem. Soc..

[12] Watanabe T., Odagi M., Furukori K., Nagasawa K. (2014). Asymmetric α-hydroxylation of a lactone with vinylogous pyridone by using a guanidine-urea bifunctional organocatalyst: Catalytic enantioselective synthesis of a key intermediate for (20S)-camptothecin analogues. Chem. Eur. J..

[13] Sim S.-B.D., Wang M., Zhao Y. (2015). Phase-transfer-catalyzed enantioselective α-hydroxylation of acyclic and cyclic ketones with oxygen. ACS Catal..

[14] Shevchenko G.A., Pupo G., List B. (2019). Direct asymmetric α-hydroxylation of cyclic α-branched ketones through enol catalysis. Synlett.

[15] Shevchenko G.A., Oppelaar B., List B. (2018). An unexpected α-oxidation of cyclic ketones with 1,4-benzoquinone by enol catalysis. Angew. Chem. Int. Ed..

[16] Shibatomi K., Kitahara K., Sasaki N., Kawasaki Y., Fujisawa I., Iwasa S. (2017). Enantioselective decarboxylative chlorination of β-ketocarboxylic acids. Nat. Commun..

[17] Masaki Y., Arasaki H., Iwata M. (2003). Stereospecific construction of chiral quaternary carbon compounds from chiral secondary alcohol derivatives. Chem. Lett..

[18] Mascal M., Hafezi N., Toney M.D. (2010). 1,4,7-Trimethyloxatriquinane: S_N_2 reaction at tertiary carbon. J. Am. Chem. Soc..

[19] Shibatomi K., Soga Y., Narayama A., Fujisawa I., Iwasa S. (2012). Highly enantioselective chlorination of β-keto esters and subsequent S_N_2 displacement of tertiary chlorides: A flexible method for the construction of quaternary stereogenic centers. J. Am. Chem. Soc..

[20] Pronin S.V., Reiher C.A., Shenvi R.A. (2013). Stereoinversion of tertiary alcohols to tertiary-alkyl isonitriles and amines. Nature.

[21] Liu R.Y., Wasa M., Jacobsen E.N. (2015). Enantioselective synthesis of tertiary α-chloro esters by non-covalent catalysis. Tetrahedron Lett..

[22] Shibatomi K., Kotozaki M., Sasaki N., Fujisawa I., Iwasa S. (2015). Williamson ether synthesis with phenols at a tertiary stereogenic carbon: Formal enantioselective phenoxylation of β-Keto esters. Chem. Eur. J..

[23] Kim D., Ha M.W., Hong S., Park C., Kim B., Yang J., Park H. (2017). Enantioselective synthesis of chiral α-azido and α-aryloxy quaternary stereogenic centers via the phase-transfer-catalyzed α-alkylation of α-bromomalonates, followed by S_N_2 Substitution. J. Org. Chem..

[24] Shibatomi K., Kitahara K., Okimi T., Abe Y., Iwasa S. (2016). Enantioselective fluorination of α-branched aldehydes and subsequent conversion to α-hydroxyacetals via stereospecific C–F bond cleavage. Chem. Sci..

[25] Ross A.G., Townsend S.D., Danishefsky S.J. (2013). Halocycloalkenones as Diels−Alder dienophiles. Applications to generating useful structural patterns. J. Org. Chem..

[26] Davis F.A., Weismiller M.C. (1990). Enantioselective synthesis of tertiary α-hydroxy carbonyl compounds using ((8,8-dichlorocamphoryl)sulfonyl)oxaziridine. J. Org. Chem..

[27] Sato K., Sekiguchi T., Aoki S. (2001). Novel methods for synthesizing 2-chloroaldehyde from carbonyl compounds and converting it into 2-hydroxyaldehyde derivatives. Tetrahedron Lett..

[28] Arai T., Sato T., Kanoh H., Kaneko K., Oguma K., Yanagisawa A. (2008). Organic-inorganic hybrid polymer-encapsulated magnetic nanobead catalysts. Chem. Eur. J..

