# Supplementary material for: Asymmetric Synthesis of Tertiary α -Hydroxyketones by Enantioselective Decarboxylative Chlorination and Subsequent Nucleophilic Substitution
Source: Molecules. 2020 Aug 27;25(17):3902. doi: 10.3390/molecules25173902 (PMC7503659; doi:10.3390/molecules25173902)
Supplement: Supplementary file 1 [file molecules-25-03902-s001.pdf]

tert-Butyl 2-(2-cyanobutyl)-1-oxo-1,2,3,4-tetrahydronaphthalene-2-carboxylate

^{1}H NMR

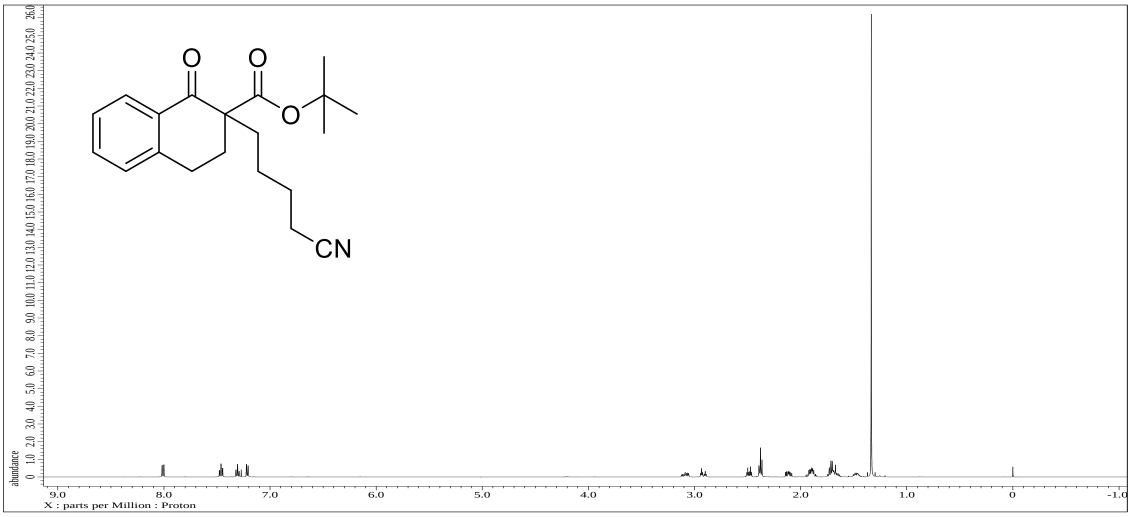

^{13}C NMR

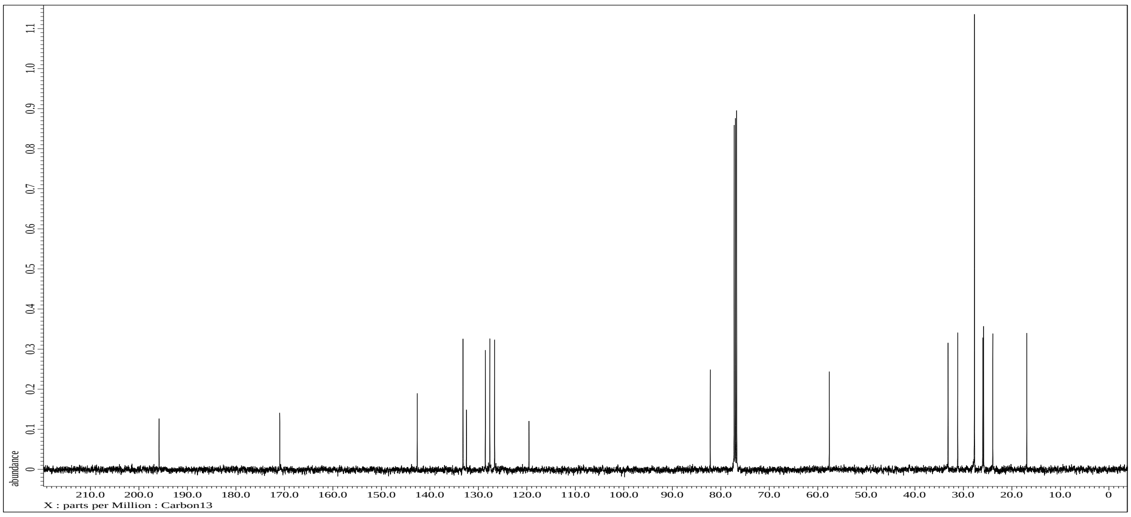

2-(4-Cyanobutyl)-1-oxo-1,2,3,4-tetrahydronaphthalene-2-carboxylic acid (1e)

^{1}H NMR

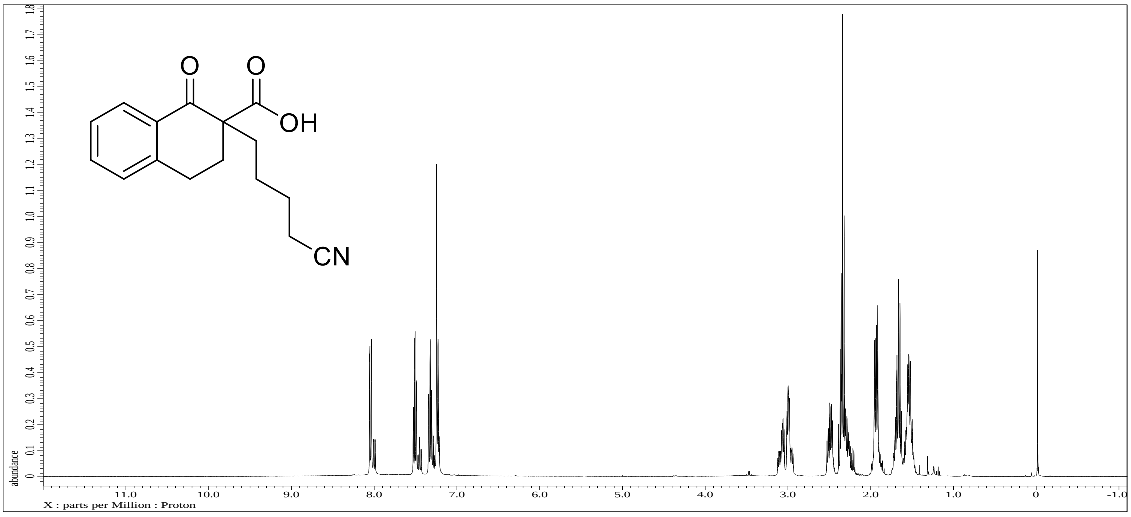

^{13}C NMR

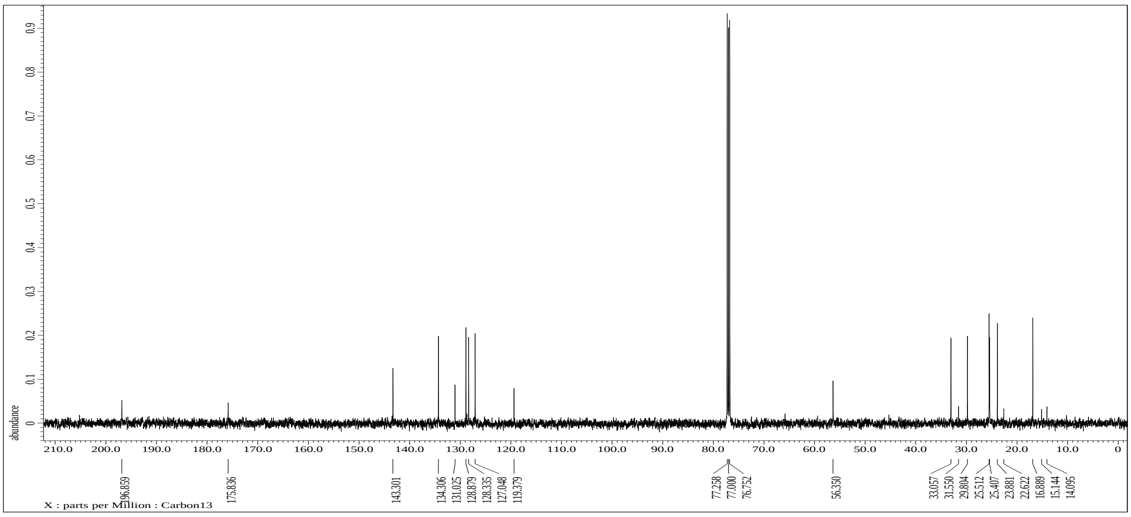

5-(2-Chloro-1-oxo-1,2,3,4-tetrahydronaphthalen-2-yl)pentanenitrile (2e)

^{1}H NMR

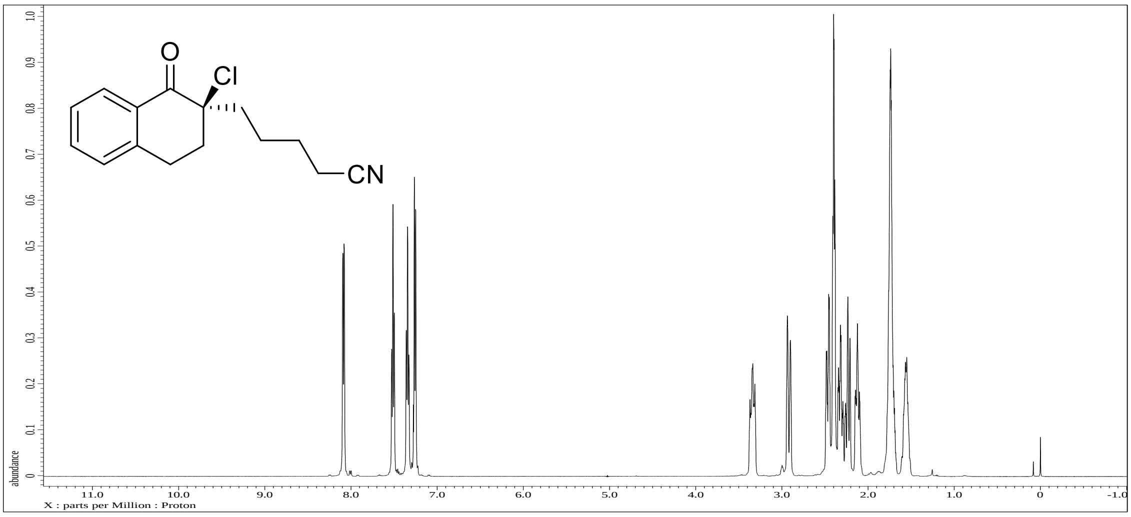

^{13}C NMR

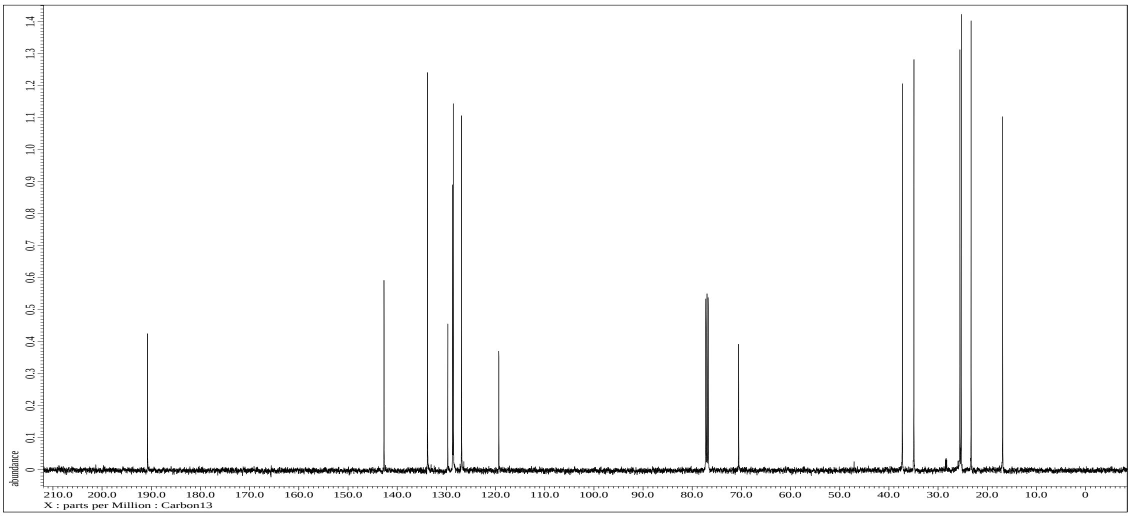

2-Allyl-2-hydroxy-3,4-dihydronaphthalen-1(2*H*)-one (3a)

^{1}H NMR

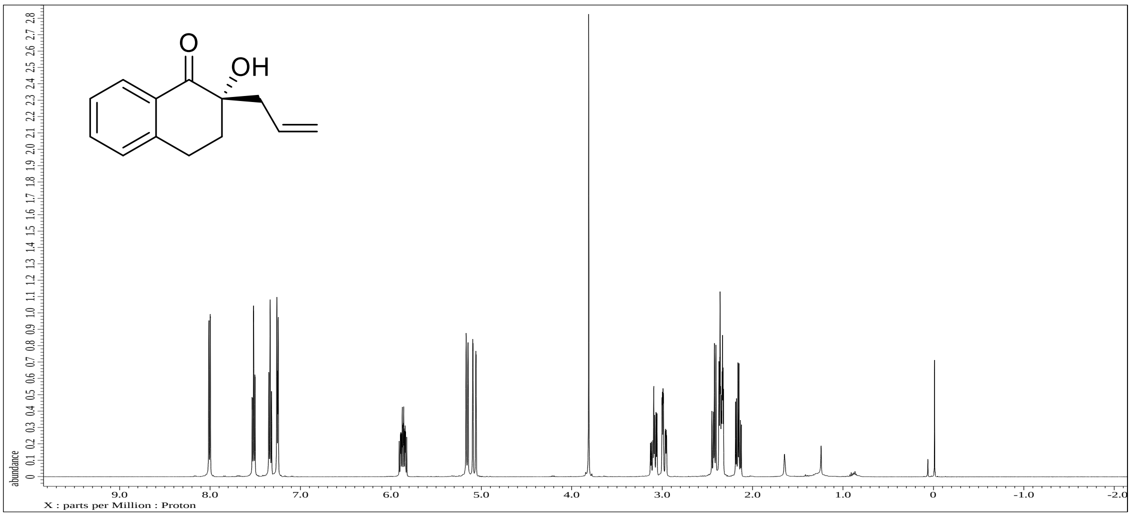

^{13}C NMR

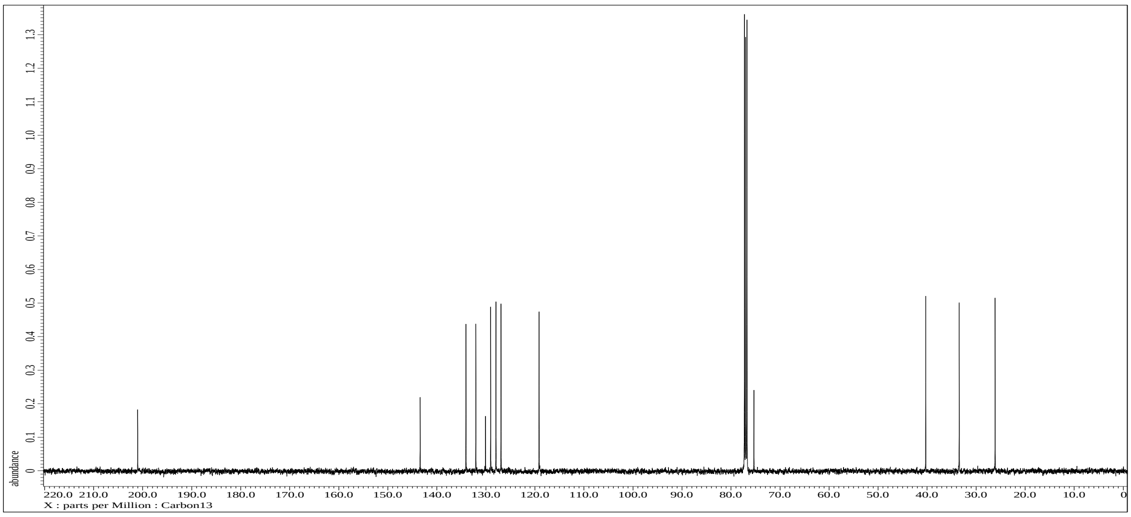

HPLC *racemic*

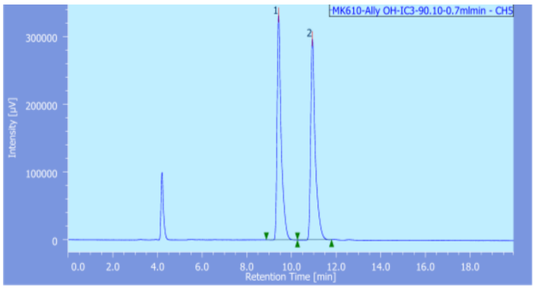

#	ピーク名	CH	tR [min]	面積 [μV·sec]	高さ [μV]	面積%	高さ%	定量値	NTP	分離度	シンメトリー係数
1	Unknown	5	9.422	4207290	331755	50.016	52.813	N/A	16110	4.828	1.723
2	Unknown	5	10.935	4204554	296418	49.984	47.187	N/A	17399	N/A	1.718

HPLC *optically active*

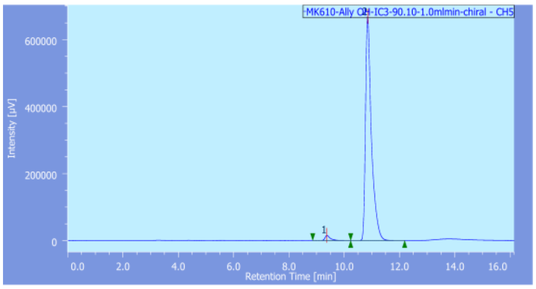

#	ピーク名	CH	tR [min]	面積 [μV·sec]	高さ [μV]	面積%	高さ%	定量値	NTP	分離度	シンメトリー係数
1	Unknown	5	9.373	211990	15654	2.075	2.285	N/A	15714	4.521	1.794
2	Unknown	5	10.845	10006818	669539	97.925	97.715	N/A	15047	N/A	1.751

(*R*)-2-Hydroxy-2-methyl-3,4-dihydronaphthalen-1(2*H*)-one (3b)

^{1}H NMR

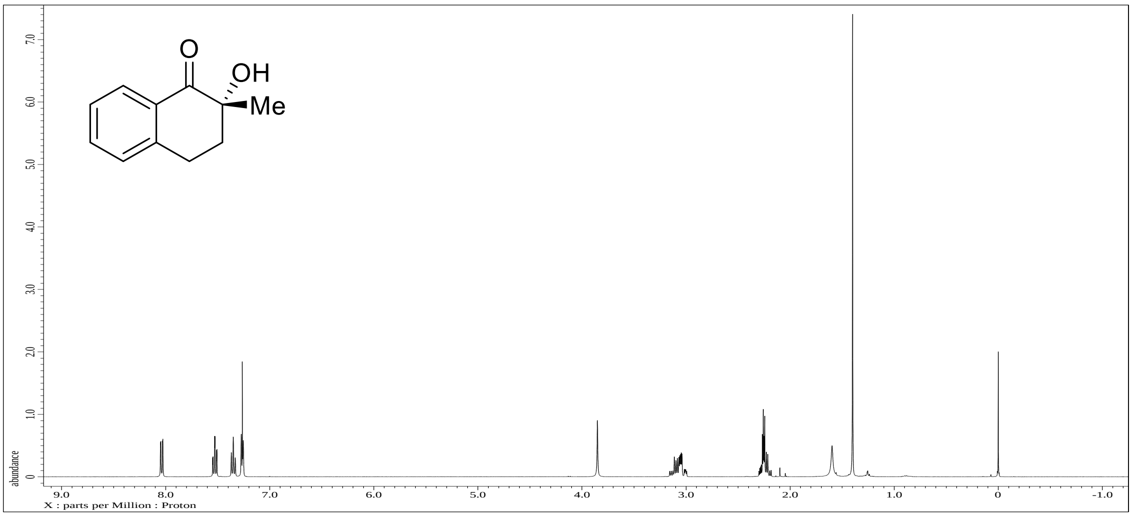

^{13}C NMR

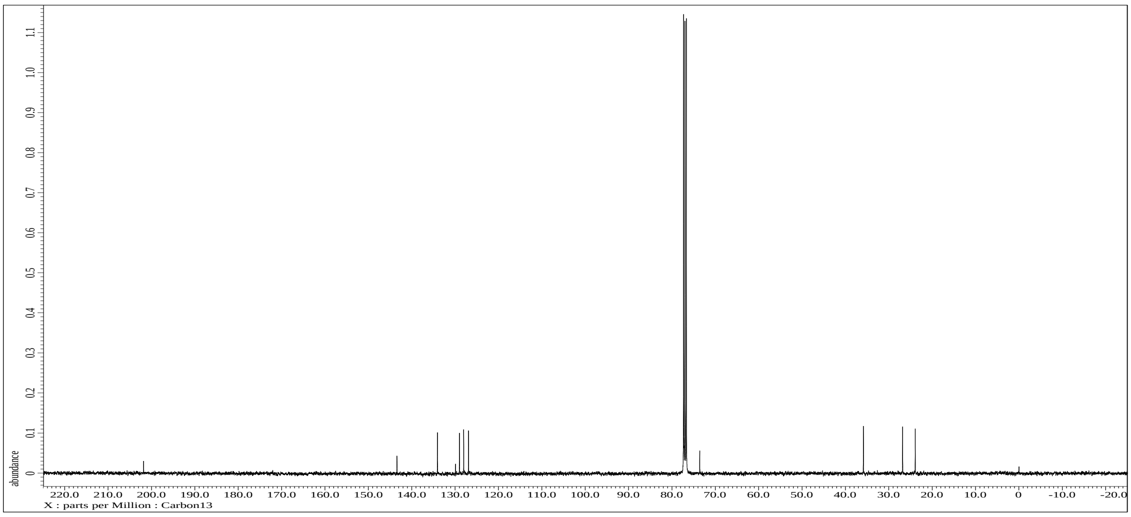

HPLC *racemic*

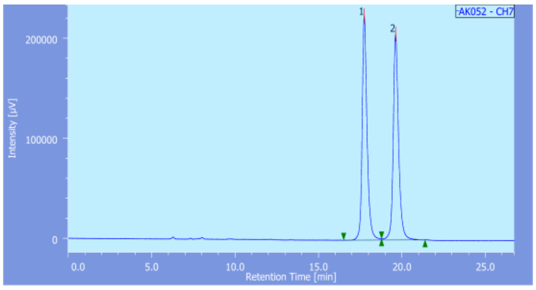

#	ピーク名	CH	tR [min]	面積 [μV·sec]	高さ [μV]	面積%	高さ%	定量値	NTP	分離度	シンメトリー係数
1	Unknown	7	17.740	4557881	224096	49.936	52.136	N/A	20670	3.647	1.264
2	Unknown	7	19.613	4569569	205737	50.064	47.864	N/A	21374	N/A	1.225

HPLC *optically active*

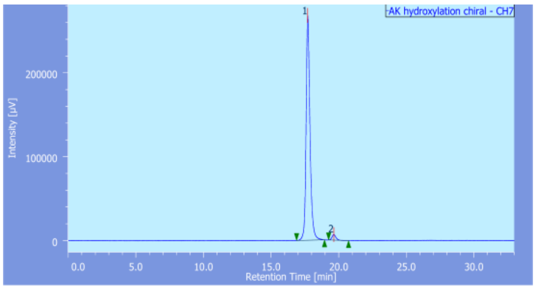

#	ピーク名	CH	tR [min]	面積 [μV·sec]	高さ [μV]	面積%	高さ%	定量値	NTP	分離度	シンメトリー係数
1	Unknown	7	17.707	5397168	267964	97.644	97.586	N/A	20919	3.904	1.257
2	Unknown	7	19.635	130224	6628	2.356	2.414	N/A	24602	N/A	1.134

2-Benzyl-2-hydroxy-3,4-dihydronaphthalen-1(2*H*)-one (3c)

^{1}H NMR

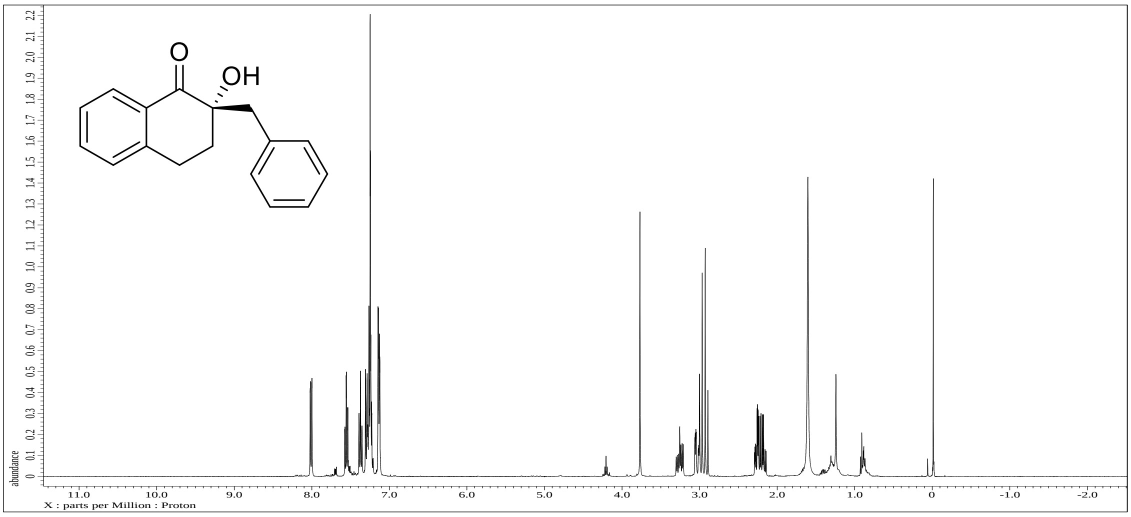

^{13}C NMR

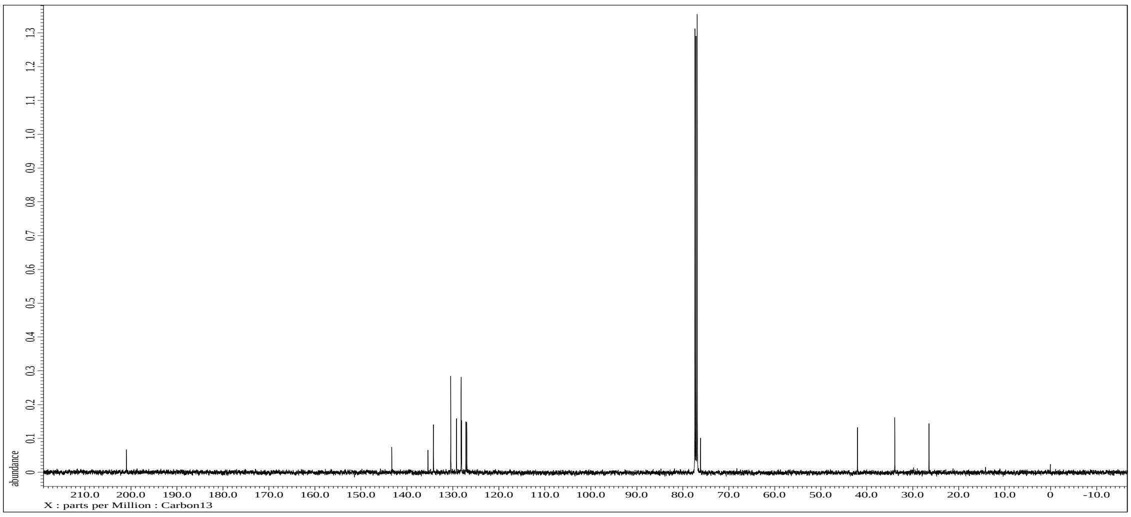

HPLC *racemic*

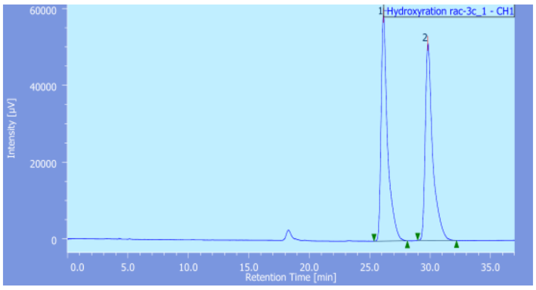

#	ピーク名	CH	tR [min]	面積 [μV·sec]	高さ [μV]	面積%	高さ%	定量値	NTP	分離度	シンメトリー係数
1	Unknown	1	26.133	2196362	58750	50.124	53.382	N/A	14996	4.036	1.832
2	Unknown	1	29.817	2185482	51306	49.876	46.618	N/A	14918	N/A	1.814

HPLC *optically active*

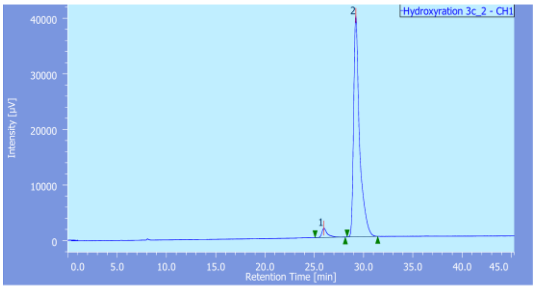

#	ピーク名	CH	tR [min]	面積 [μV·sec]	高さ [μV]	面積%	高さ%	定量値	NTP	分離度	シンメトリー係数
1	Unknown	1	25.967	63483	1690	3.679	4.070	N/A	15046	3.617	1.771
2	Unknown	1	29.208	1662012	39828	96.321	95.930	N/A	15110	N/A	1.811

3-(2-Hydroxy-1-oxo-1,2,3,4-tetrahydronaphthalen-2-yl)propanenitrile (3d)

^{1}H NMR

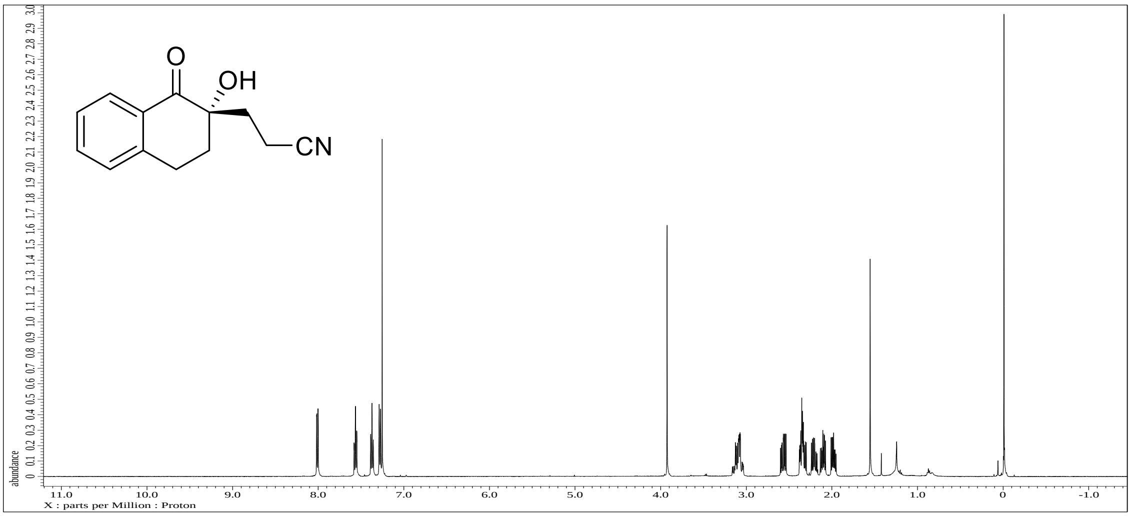

^{13}C NMR

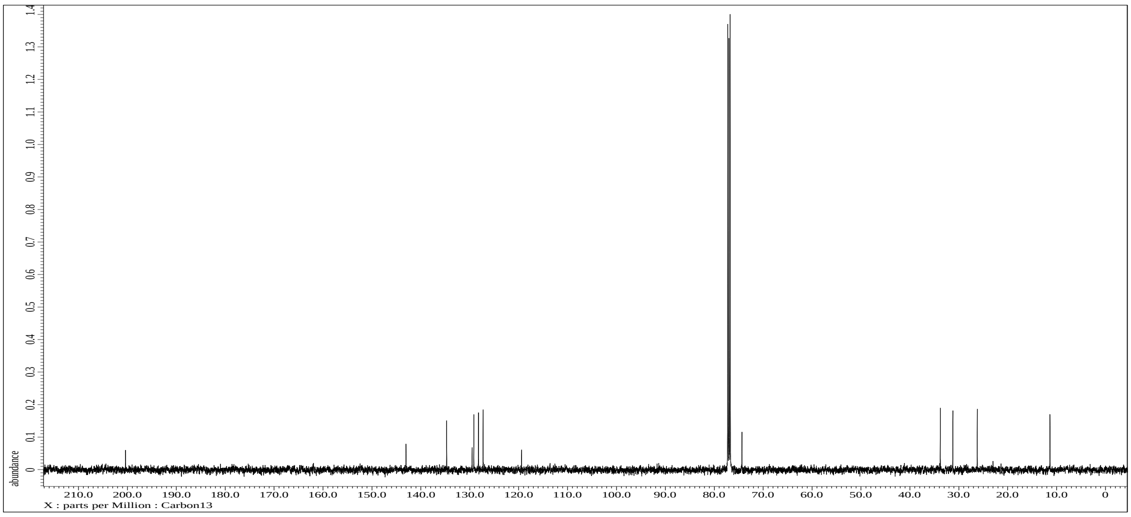

HPLC *racemic*

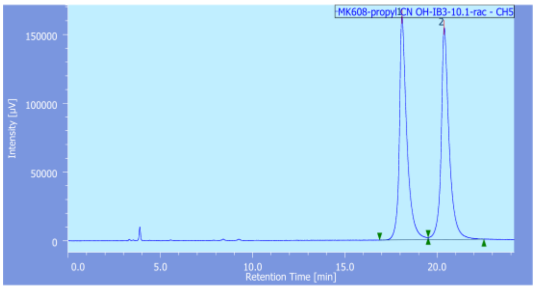

#	ピーク名	CH	tR [min]	面積 [µV·sec]	高さ [µV]	面積%	高さ%	定量値	NTP	分離度	シンメトリー係数
1	Unknown	5	18.095	4604654	162603	49.785	51.342	N/A	12597	3.451	1.454
2	Unknown	5	20.382	4644433	154102	50.215	48.658	N/A	14212	N/A	1.296

HPLC *optically active*

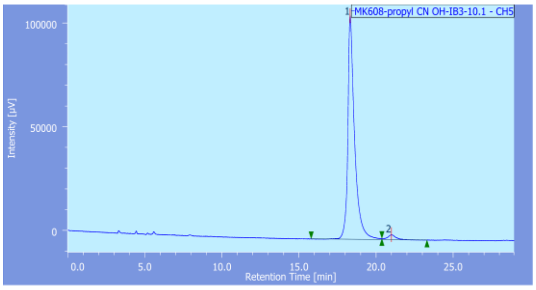

#	ピーク名	CH	tR [min]	面積 [µV·sec]	高さ [µV]	面積%	高さ%	定量値	NTP	分離度	シンメトリー係数
1	Unknown	5	18.313	3289528	107782	97.321	97.840	N/A	11416	3.522	1.531
2	Unknown	5	20.992	90541	2380	2.679	2.160	N/A	10008	N/A	N/A

5-(2-Hydroxy-1-oxo-1,2,3,4-tetrahydronaphthalen-2-yl)pentanenitrile (3e)

^{1}H NMR

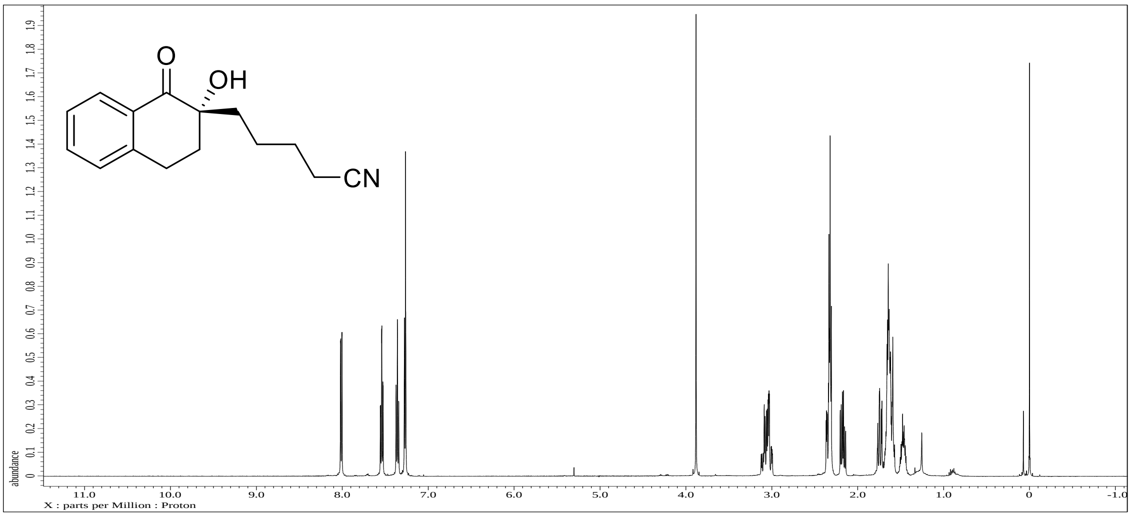

^{13}C NMR

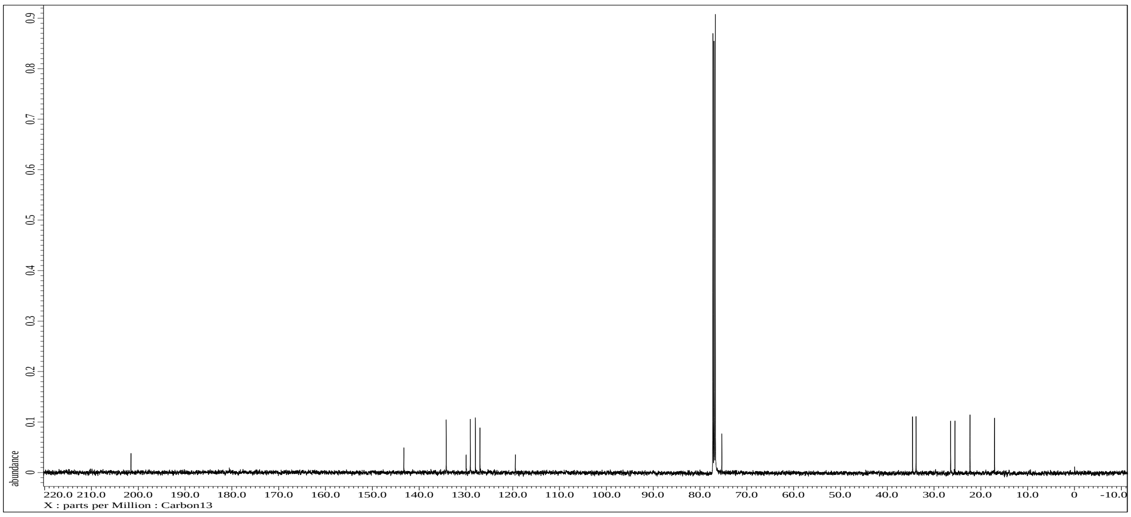

HPLC *racemic*

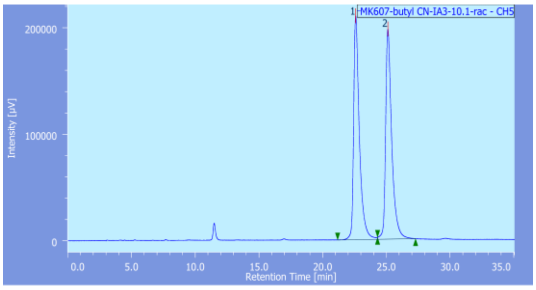

#	ピーク名	CH	tR [min]	面積 [µV·sec]	高さ [µV]	面積%	高さ%	定量値	NTP	分離度	シンメトリー係数
1	Unknown	5	22.587	6545780	210079	49.987	51.577	N/A	14830	3.300	1.710
2	Unknown	5	25.113	6549126	197230	50.013	48.423	N/A	16028	N/A	1.614

HPLC *optically active*

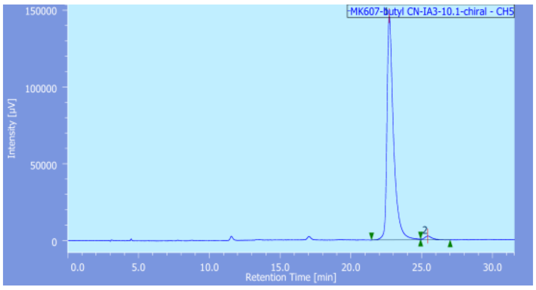

#	ピーク名	CH	tR [min]	面積 [µV·sec]	高さ [µV]	面積%	高さ%	定量値	NTP	分離度	シンメトリー係数
1	Unknown	5	22.707	4692015	145947	97.991	98.249	N/A	14268	3.310	1.737
2	Unknown	5	25.432	96196	2601	2.009	1.751	N/A	13050	N/A	N/A

6-Chloro-3-hydroxy-3-methylchroman-4-one (3f)

^{1}H NMR

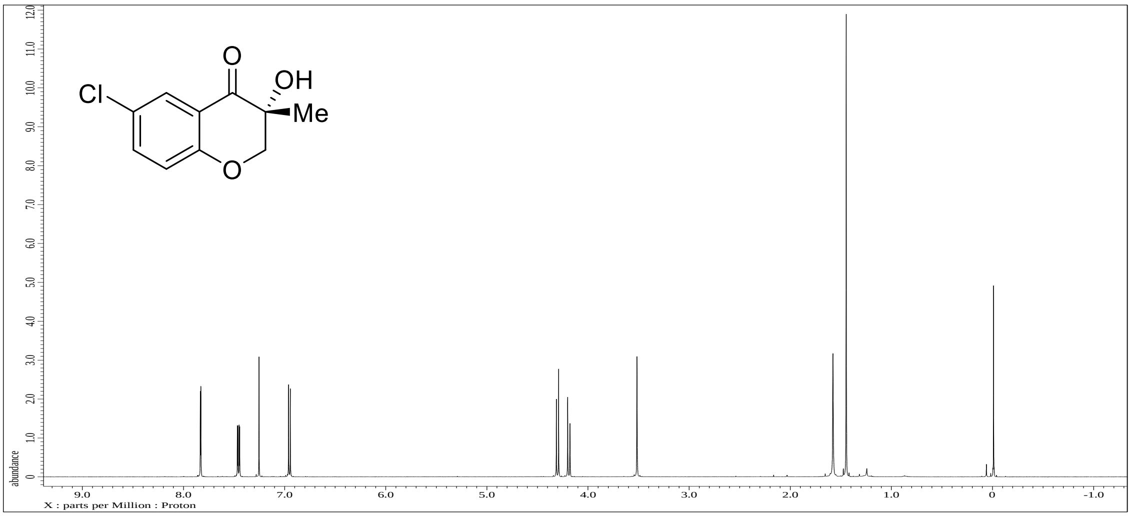

^{13}C NMR

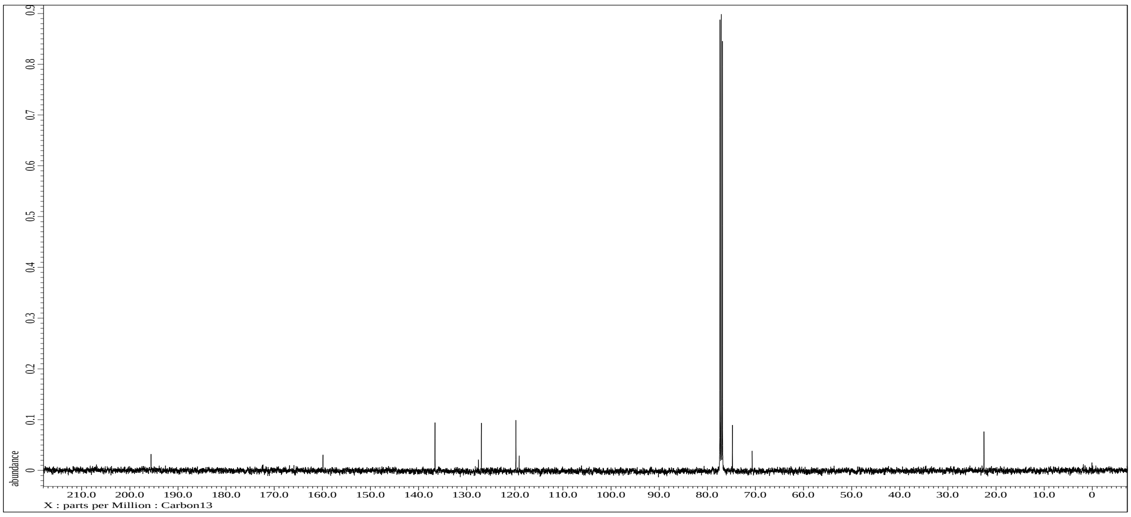

HPLC *racemic*

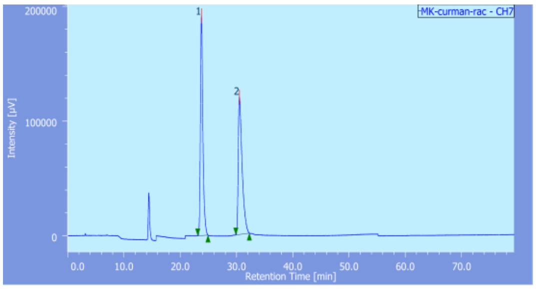

#	ピーク名	CH	tR [min]	面積 [µV·sec]	高さ [µV]	面積%	高さ%	定量値	NTP	分離度	シンメトリー係数
1	Unknown	7	23.765	5820034	191511	50.563	61.513	N/A	16048	6.961	1.385
2	Unknown	7	30.512	5690404	119824	49.437	38.487	N/A	10463	N/A	2.003

HPLC *optically active*

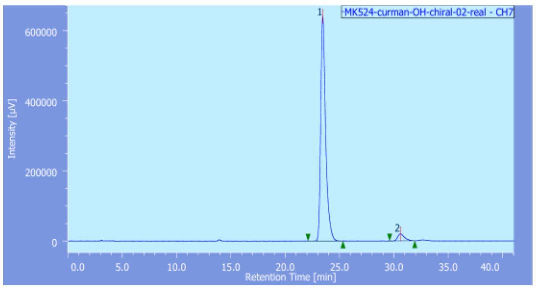

#	ピーク名	CH	tR [min]	面積 [µV·sec]	高さ [µV]	面積%	高さ%	定量値	NTP	分離度	シンメトリー係数
1	Unknown	7	23.447	20157586	639862	95.897	96.935	N/A	14546	7.672	1.487
2	Unknown	7	30.592	862471	20230	4.103	3.065	N/A	12604	N/A	1.461

2-Hydroxy-2-methyl-2,3-dihydro-1*H*-inden-1-one (3g)

^{1}H NMR

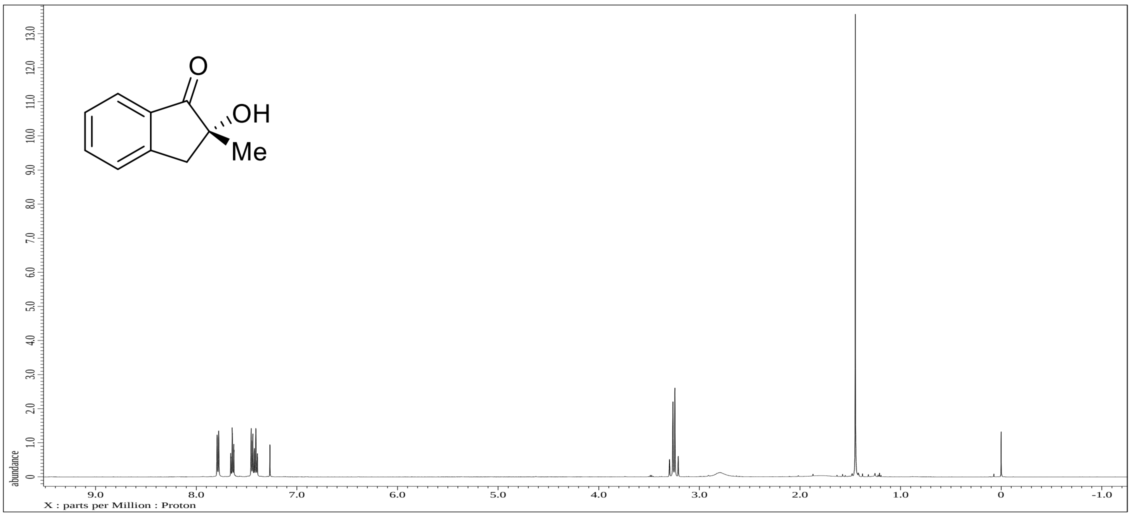

^{13}C NMR

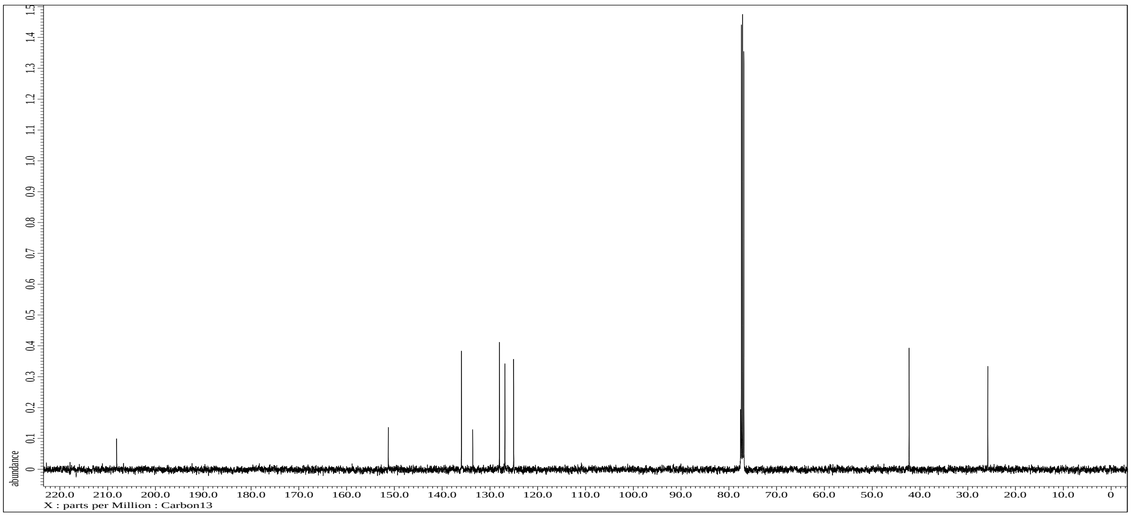

HPLC *racemic*

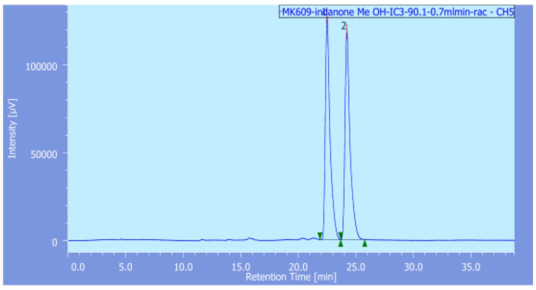

#	ピーク名	CH	tR [min]	面積 [μV·sec]	高さ [μV]	面積%	高さ%	定量値	NTP	分離度	シンメトリー係数
1	Unknown	5	22.490	3626523	127113	49.853	51.771	N/A	17853	2.462	1.684
2	Unknown	5	24.207	3647856	118415	50.147	48.229	N/A	17827	N/A	1.704

HPLC *optically active*

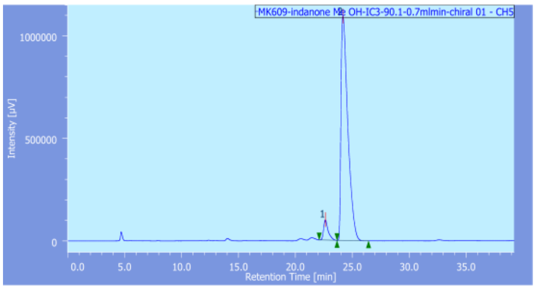

#	ピーク名	CH	tR [min]	面積 [μV·sec]	高さ [μV]	面積%	高さ%	定量値	NTP	分離度	シンメトリー係数
1	Unknown	5	22.628	2732476	99159	5.668	8.327	N/A	18713	1.788	1.570
2	Unknown	5	24.172	45473858	1091686	94.332	91.673	N/A	8170	N/A	2.281

2-Benyl-2-hydroxycyclohexan-1-one (3h)

^{1}H NMR

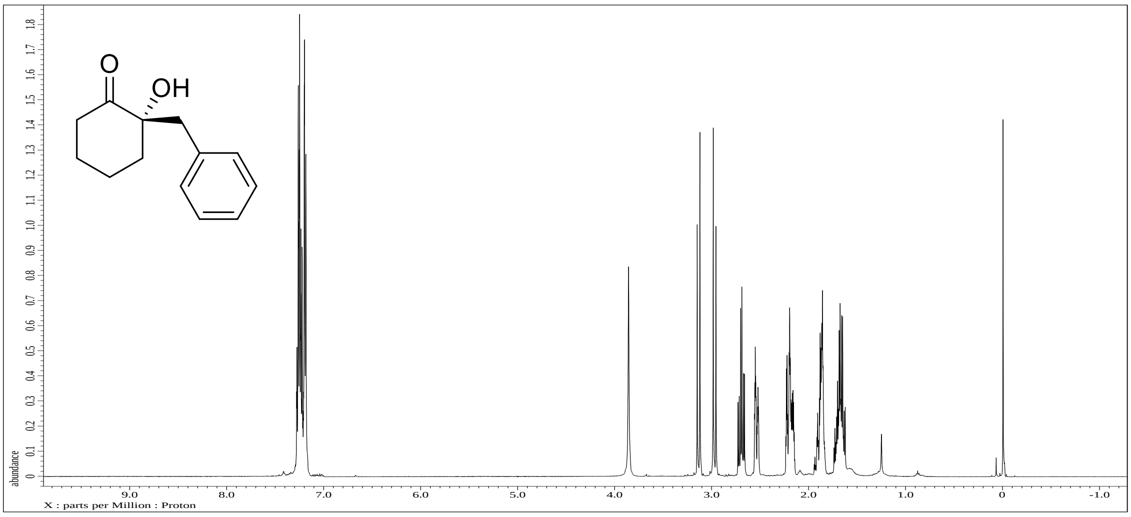

^{13}C NMR

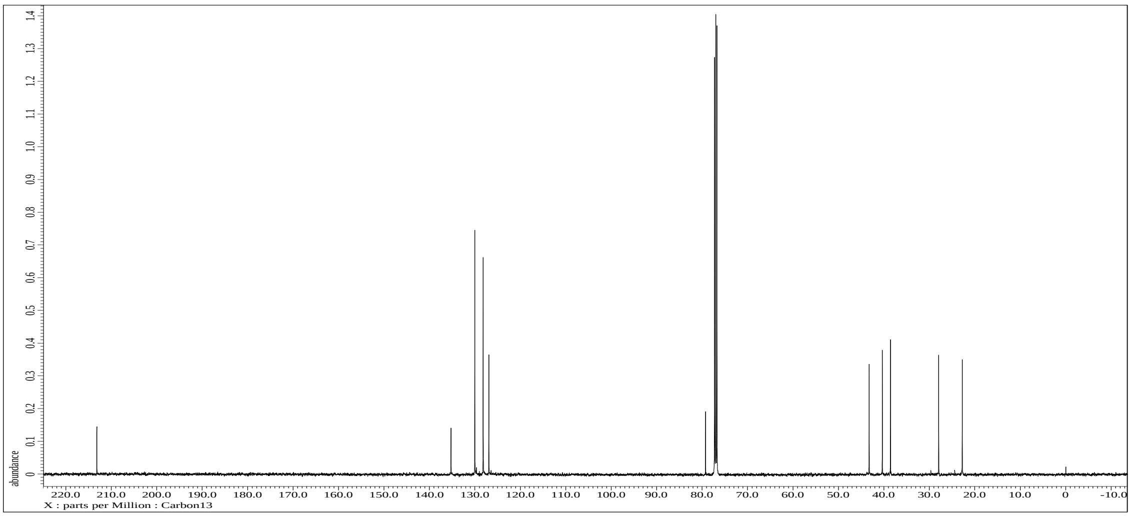

HPLC *racemic*

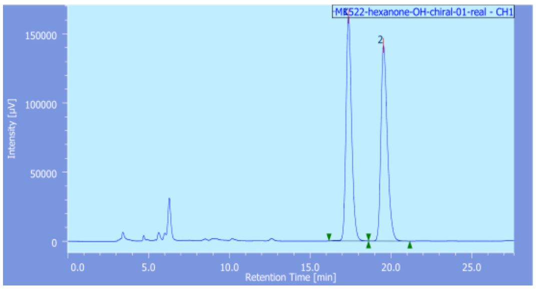

#	ピーク名	CH	tR [min]	面積 [µV·sec]	高さ [µV]	面積%	高さ%	定量値	NTP	分離度	シンメトリー係数
1	Unknown	1	17.367	3928166	162453	50.042	53.462	N/A	12476	3.249	1.240
2	Unknown	1	19.533	3921614	141414	49.958	46.538	N/A	11925	N/A	1.368

HPLC *optically active*

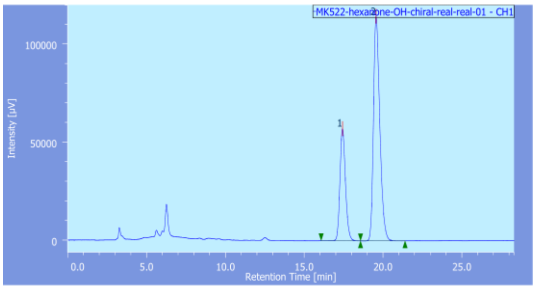

#	ピーク名	CH	tR [min]	面積 [µV·sec]	高さ [µV]	面積%	高さ%	定量値	NTP	分離度	シンメトリー係数
1	Unknown	1	17.425	1382718	57110	30.133	33.287	N/A	12428	3.170	1.171
2	Unknown	1	19.558	3205981	114457	69.867	66.713	N/A	11669	N/A	1.316
